# Lytic viruses drive the decrease in polyphosphate-accumulating and phosphate-solubilizing potential of microbial communities with increasing reservoir age

**DOI:** 10.1128/aem.02481-25

**Published:** 2026-04-27

**Authors:** Qiusheng Wu, Debin Wu, Jiayi Wang, Heng Wang, Jingjing Peng, Yuan Zhao, Jingan Chen, Quan Yuan

**Affiliations:** 1 State Key Laboratory of Environment Geochemistry, Institute of Geochemistry, Chinese Academy of Sciences 85408 , Guiyang, China; 2 Guizhou Province Field Scientific Observation and Research Station of Hongfeng Lake Reservoir Ecosystem, Guiyang, China; 3 University of Chinese Academy of Sciences 74519, Beijing, China; 4 State Key Laboratory of Nutrient Use and Management, College of Resources and Environmental Sciences, China Agricultural University 34752, Beijing, China; University of Delaware, Lewes, Delaware, USA

**Keywords:** damming, reservoir age, P-cycling functional gene, viral diversity, auxiliary metabolic genes

## Abstract

River damming often leads to significant phosphorus enrichment in reservoir sediments and increases the risk of eutrophication with reservoir age. Microorganisms mediate critical steps of phosphorus cycling in ecosystems, and viruses are recognized as key regulators of microbial community structure and function. However, their influence on phosphorus-cycling microorganisms (PCMs) in freshwater environments remains poorly understood. In this study, surface sediment samples were collected from nine reservoirs (12–59 years old) of southwest China and analyzed using metagenomic and metatranscriptomic approaches to profile both PCMs and viral communities. The results demonstrated that the diversity of lytic viruses was the primary factor governing both shifts in the community stability of PCMs and the restructuring of P-cycling gene patterns with increasing reservoir age. Specifically, viral lysis reduced the relative abundance of dominant PCMs, thereby enhancing community diversity and stability. Concurrently, viral activity diminished PCMs’ functional potential for phosphate solubilization and polyphosphate accumulation, while stimulating high-affinity inorganic phosphate (Pi) transport. Furthermore, viruses encoded auxiliary metabolic genes (AMGs) related to phosphate solubilization, mineralization, accumulation, and transport, underscoring the viral role in regulating phosphorus retention and release. Compared to polyphosphate-accumulating microorganisms, phosphate-solubilizing microorganisms may be more susceptible to viral infection. Additionally, viral activity was associated with an increase in the relative abundance of Cyanobacteria. Taken together, our results suggest viruses are key regulators of PCMs, highlighting that they should be incorporated into future strategies for assessing and mitigating reservoir eutrophication.

**IMPORTANCE:**

Sediment microorganisms are regarded as the engine for endogenous phosphorus release in reservoirs. Therefore, understanding their dynamics and key driving factors is essential for effective eutrophication mitigation. Viral lysis and virus-encoded auxiliary metabolic genes (AMGs) may constitute a critical yet understudied mechanism influencing microbial phosphorus cycling. Our study provides unique, time-series-based mechanistic insights into how viral activity, in the context of large-scale artificial projects (river damming), restructures microbial phosphorus cycling and its potential ecological effects over decades.

## INTRODUCTION

To alleviate the increasingly severe water and energy shortages, numerous hydroelectric dams have been constructed worldwide. Consequently, the number of large dams has exceeded 70,000 globally (1). Studies have shown that river damming traps significant quantities of phosphorus in reservoir sediments, substantially reducing its downstream transport along the river continuum (2–4). This accumulation of phosphorus causes sediments to act as both a source and a sink of phosphorus for the overlying water (5, 6), thereby increasing the risk of eutrophication and algal blooms. Understanding the mechanisms of phosphorus cycling in reservoir sediments is therefore essential for developing effective eutrophication mitigation strategies.

Microorganisms play a pivotal role in the biogeochemical cycling of phosphorus by harboring P-cycling genes (7–9). These P-cycling microorganisms (PCMs) mediate key processes including polyphosphate accumulation (PA, e.g., *ppk1* and *ppk2*), inorganic phosphate solubilization (IPS, e.g., *gcd* and *pqqABCDE*), organic phosphate mineralization (OPM, e.g., *phoA, phoC* and *phoD*), phosphate transport (e.g., *pit* and *pstSABC*), and phosphate-starvation response regulation (e.g., *phoH*) (10–13). The process of PA involves the reversible synthesis of inorganic polyphosphate (poly P) from the terminal phosphate of ATP or GTP, catalyzed by poly P kinase (encoded by *ppk1* or *ppk2*) (13). Therefore, polyphosphate-accumulating microorganisms (PAMs) play a central role in enhanced biological phosphorus removal (14). For IPS and OPM, microorganisms secrete organic acids and enzymes that convert recalcitrant phosphorus into a bioavailable state, ultimately elevating bioavailable phosphorus content in both soils and oceans (15–17). These processes are considered potential drivers of endogenous phosphorus release from sediments (18). Additionally, PCMs perceive bioavailable phosphorus via starvation-response regulators and mediate its uptake through high-affinity (*pstABCS*) and low-affinity (*pit*) transporters (19, 20). In freshwater ecosystems, dam construction has been shown to enhance alkaline phosphatase activity in sediments (21), which can promote phosphorus release from sediments by phosphate-solubilizing microorganisms (PSMs) and thereby increase eutrophication risks (22). Previous work has demonstrated a strong link between carbon cycling in sediments and reservoir age (23). However, it remains unclear how the community structure of PCMs and P-cycling functional genes responds to changes in reservoir age.

Viruses are the most abundant biological entities on Earth, with an estimated 4.8 × 10^³¹^ particles (24). Previous research has shown that viruses shape the structure and function of microbial communities through two primary mechanisms: the lysis of host cells and the carriage of diverse auxiliary metabolic genes (AMGs) that reprogram or enhance host metabolism (25–27). These processes play a critical role in nutrient cycling across both aquatic and terrestrial ecosystems (28, 29). For example, viral lysis in soils enhances organic matter storage and alleviates phosphorus limitation (30), while the expression of AMGs, such as those involved in carbon fixation and the phosphate-starvation response, improves microbial carbon sequestration and adaptation to phosphorus deficiency (31, 32). Microbial-mediated phosphorus cycling is a key driver of eutrophication and algal blooms in freshwater environments (33, 34). However, as the reservoir age increases, it remains poorly understood how viral lysis remodels the community structure of PCMs and alters the abundance of P-cycling genes. An equally critical unanswered question is how the expression of virus-encoded P-cycling AMGs influences the diversity of PCMs with increasing reservoir age.

To unveil the knowledge gaps mentioned above, we chose reservoirs located in the Wujiang River basin as our study sites. Sediment samples were collected from reservoirs of different ages, and then both metagenomic and metatranscriptomic sequencing were conducted. Here, we aimed to (i) delineate the change of P-cycling genes as well as the diversity of PCMs and viruses with increasing reservoir age and (ii) decipher potential mechanisms underlying viral modulation of microbial P-cycling strategies. Our study might shed light on the significant impact of viruses on the populations and functions of PCMs in reservoirs, thereby providing a new perspective for managing eutrophication in reservoir ecosystems.

## RESULTS

### The chemical parameters of sediment samples

**Fig 1:**
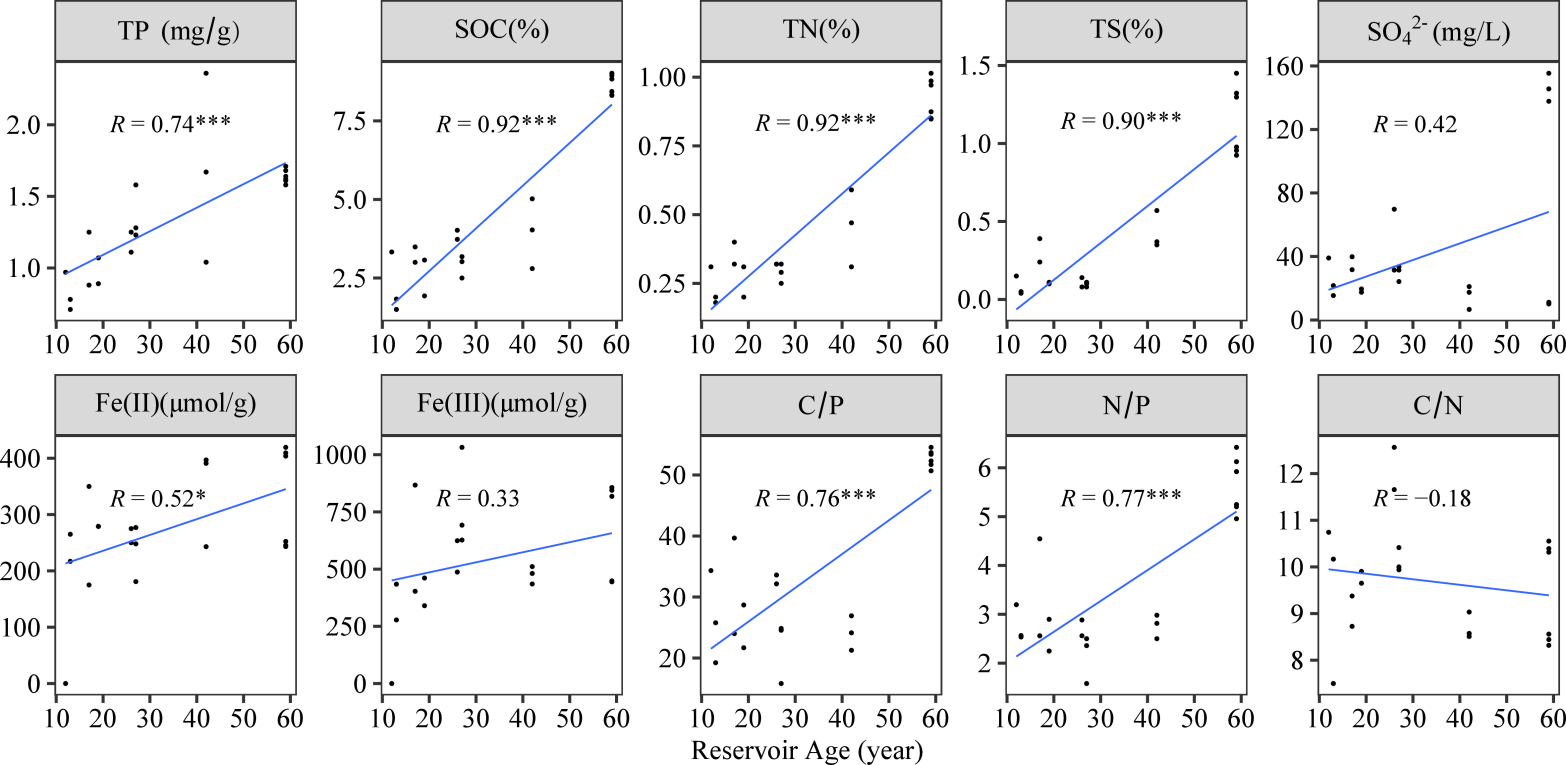
The correlation between physicochemical parameters and reservoir age in sediments. Note: *, **, and *** indicate significant differences at *P* < 0.05, *P* < 0.01, and *P* < 0.001, respectively. And apply to all subsequent figures.

In this study, the content of total phosphorus (TP) in reservoir sediments ranged from 0.71 to 2.36 mg g⁻¹, sediment organic carbon (SOC) from 1.50% to 9.02 %, and total nitrogen (TN) from 0.18% to 1.01 % (Fig. 1). All three parameters exhibited significant positive correlations with reservoir age. C/P, N/P, total sulfur (TS), and Fe(II) also increased significantly with reservoir age (Fig. 1). In contrast, SO_4_^2−^, Fe(III), and C/N showed no significant relationship with reservoir age.

### P-cycling gene abundances and microbial community profiles

**Fig 2:**
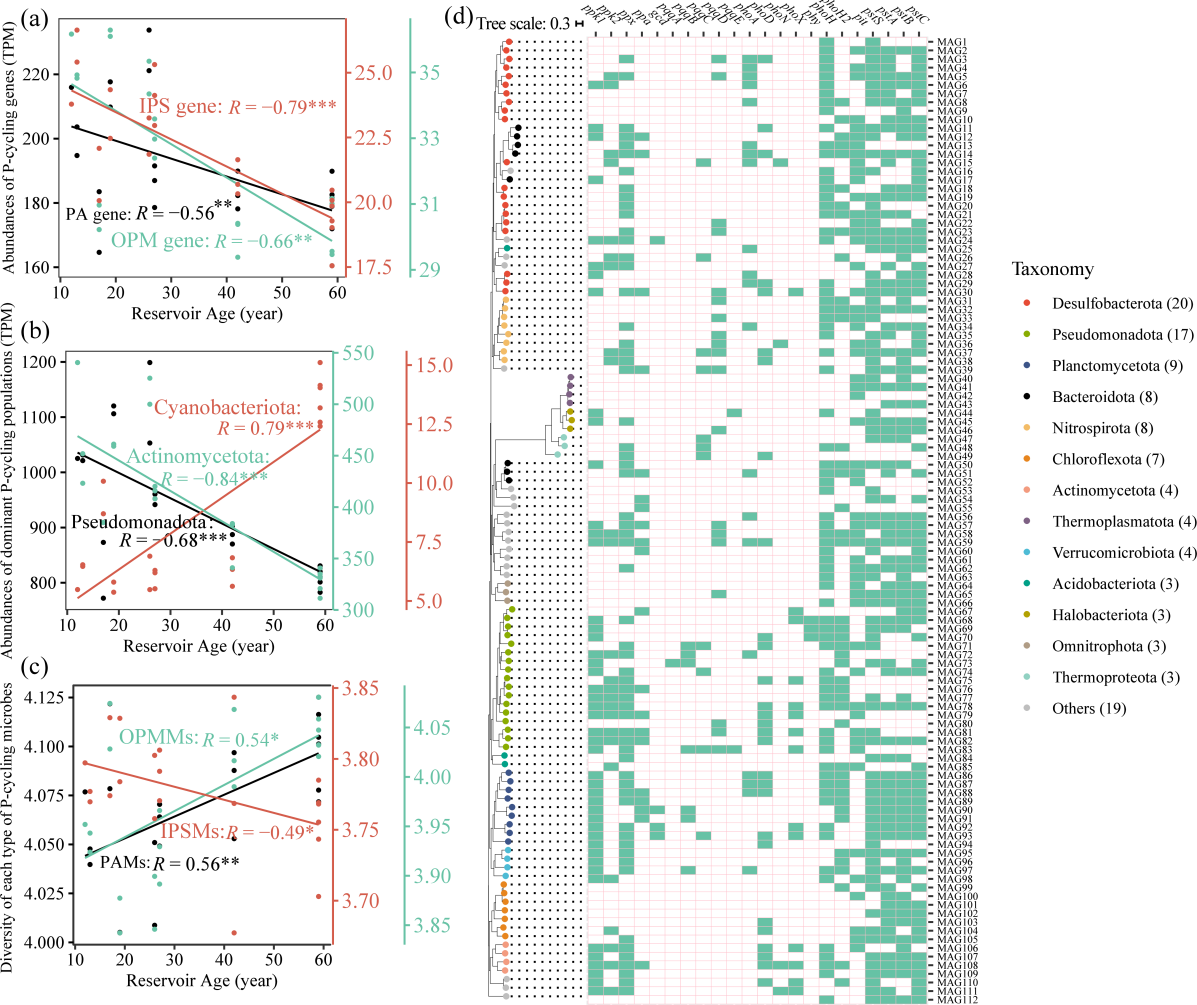
The correlations of reservoir age with P-cycling pathways and associated microbial guilds. Correlations of PA, IPS, and OPM gene abundances with reservoir age (**a**). Shifts in dominant PCMs taxa at the phylum level with increasing reservoir age (**b**). Relationships between reservoir age and the diversity of PAMs, inorganic phosphate-solubilizing microorganisms (IPSMs), and organic phosphate-mineralizing microorganisms (OPMMs; **c**). Phylogenetic and functional characterization of 112 high-quality MAGs recovered from sediments (**d**). The presence (colored) and absence (blank) of P-cycling genes are represented by the heatmap.

The gene abundances of the PA, IPS, and OPM pathways were significantly negatively correlated with reservoir age (Fig. 2a). The phosphonate biosynthetic gene, the *pepM,* and the low-affinity inorganic phosphate transporter gene, the *pit,* were both significantly negatively correlated with reservoir age (Fig. S1a and b), whereas the *pstS* was significantly positively correlated with reservoir age (Fig. S1c). Among the phosphate-starvation-response regulatory genes, *phoH2* and *phoR* were significantly positively correlated with reservoir age (Fig. S1d and f), while *phoB* was negatively correlated (Fig. S1e). The gene abundance of the PA pathway exceeded that of the OPM or the IPS pathway (Fig. S2a).

Among PCMs, the 10 most abundant phyla included Pseudomonadota, Actinomycetota, and Cyanobacteriota (Fig. S3a). The abundances of the first two phyla were significantly negatively correlated with reservoir age, whereas Cyanobacteriota was significantly positively correlated (Fig. 2b). The 10 most abundant genera comprised *Streptomyces*, *Pseudomonas*, *Burkholderia*, *Bradyrhizobium*, and *Cupriavidus* (Fig. S3b), and the diversity of these PCMs was significantly positively correlated with reservoir age (Fig. S3c). Specifically, *Pseudomonas*, *Streptomyces*, *Bradyrhizobium*, *Cupriavidus*, and *Burkholderia* were the dominant genera in PAMs, IPSMs, and OPMMs (Fig. S3d through f). The diversities of the PAMs and OPMMs were each significantly positively correlated with reservoir age (Fig. 2c), whereas that of the IPSMs was significantly negatively correlated (Fig. 2c). The diversity of the PAMs was greater than that of the OPMMs and IPSMs (Fig. S2b). Metatranscriptomic data revealed that Pseudomonadota and Actinomycetota exhibited the highest expression activities at the phylum level, and Cyanobacteriota ranked within the top 15 (Fig. S4a). At the genus level, *Pseudomonas*, *Burkholderia*, *Bradyrhizobium*, and *Streptomyces* ranked among the top 15 in expression activity (Fig. S4b).

Through binning and functional annotation, we obtained 112 bacterial and archaeal metagenome-assembled genomes (MAGs) harboring genes involved in the P cycle (Fig. 2d; Table S3). Specifically, 55 MAGs contained polyphosphate accumulation genes (*ppk1* and/or *ppk2*), including those affiliated with *Luteolibacter* (MAG95 and MAG96), *Cyanobium* (MAG110 and MAG111), and the archaeon *Methanoregula* (MAG45 and MAG46). For inorganic phosphate solubilization, the genes *pqqC* and *pqqD* were identified in 11 and 19 MAGs, respectively. These included MAGs assigned to the archaeal genera *Methanothrix* (MAG46) and *Nitrosarchaeum* (MAG47 and MAG48). Regarding organic phosphorus mineralization, the genes *phoA* and *phoD* were detected in 41 MAGs, including a MAG within the archaeal family *Nitrososphaeraceae* (MAG49). For inorganic phosphate transport, the *pit* gene was found in 54 MAGs, while the *pstSABC* operon was present in 101 MAGs, which included a MAG from the phylum Patescibacteriota (MAG54).

### Co-occurrence network of PCMs

**Fig 3:**
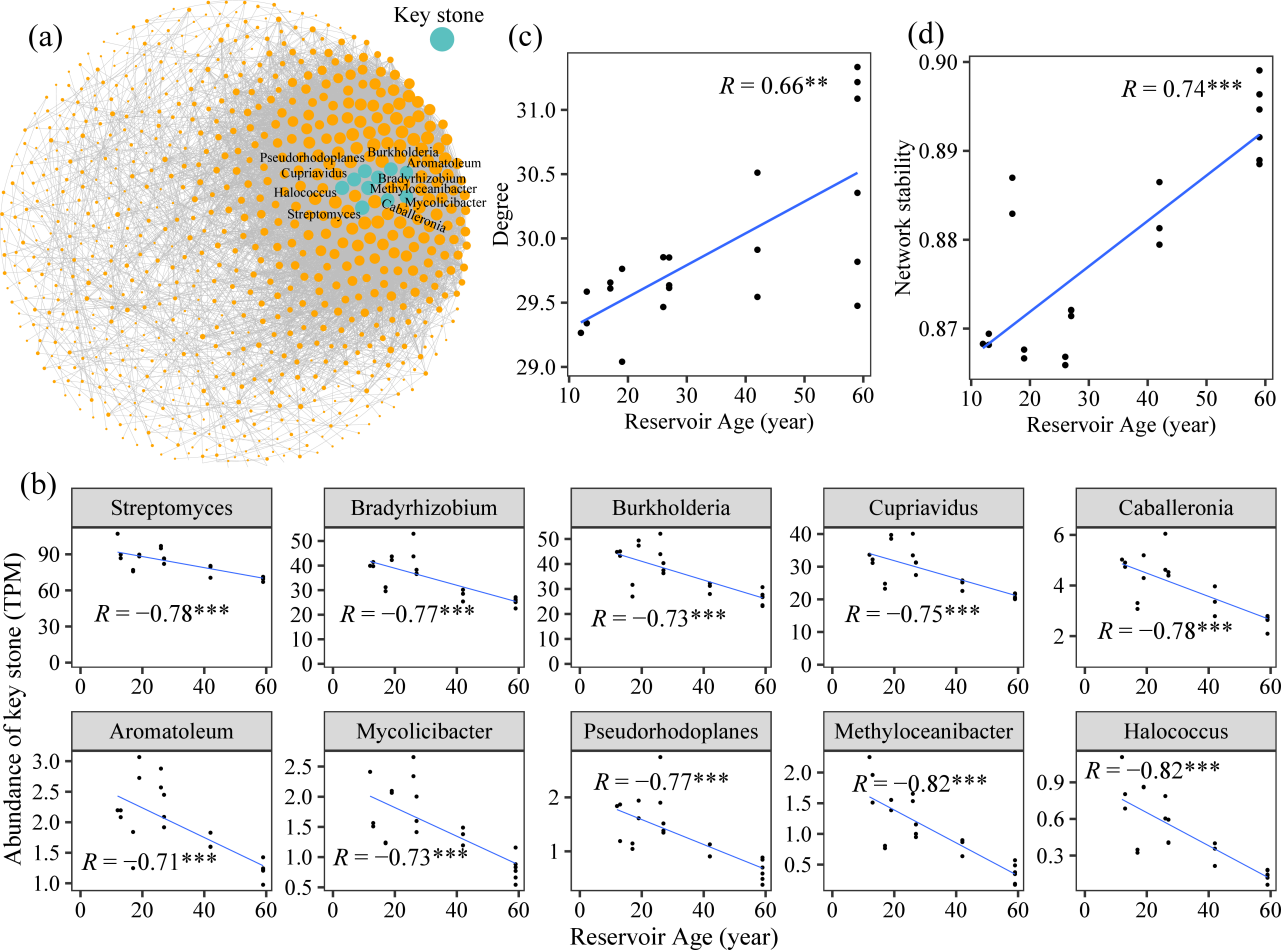
Genus-level co-occurrence network and topological features of PCMs. The 10 genera with the highest degree in the co-occurrence network (**a**). Correlations between the abundance of these ten genera and reservoir age (**b**). Relationships between key topological parameters of the network and reservoir age (**c and d**).

A co-occurrence network of PCMs was constructed at the genus level (Fig. 3a). All the 10 highest-degree taxa, including *Streptomyces*, *Bradyrhizobium*, *Cupriavidus*, and *Mycolicibacter,* were significantly negatively correlated with reservoir age (Fig. 3b). Sub-networks were extracted from the global network, and the degree of each sub-network was calculated; the sub-network degree was significantly positively correlated with reservoir age (Fig. 3c). Positive cohesion within the sub-networks decreased significantly along the reservoir-age gradient, whereas negative cohesion increased significantly (Fig. S5a and b). Network stability also increased significantly along the same pattern (Fig. 3d).

### Viral community, host prediction, and AMG profiles

A total of 13,533 non-redundant viral operational taxonomic units (vOTUs) were identified from the metagenomes. Compared with the RefSeq viral genomes, only 13.38% of these vOTUs shared proteins with the database, leaving 86.62% of the vOTUs unique to the present sediments (Fig. S6a and b). Taxonomic annotation indicated that 58.86% of the vOTUs were assigned to Uroviricota, 10.42% to Nucleocytoviricota, 0.68% to Preplasmiviricota, and 29.46% remained unclassified (Fig. S6b).

**Fig 4:**
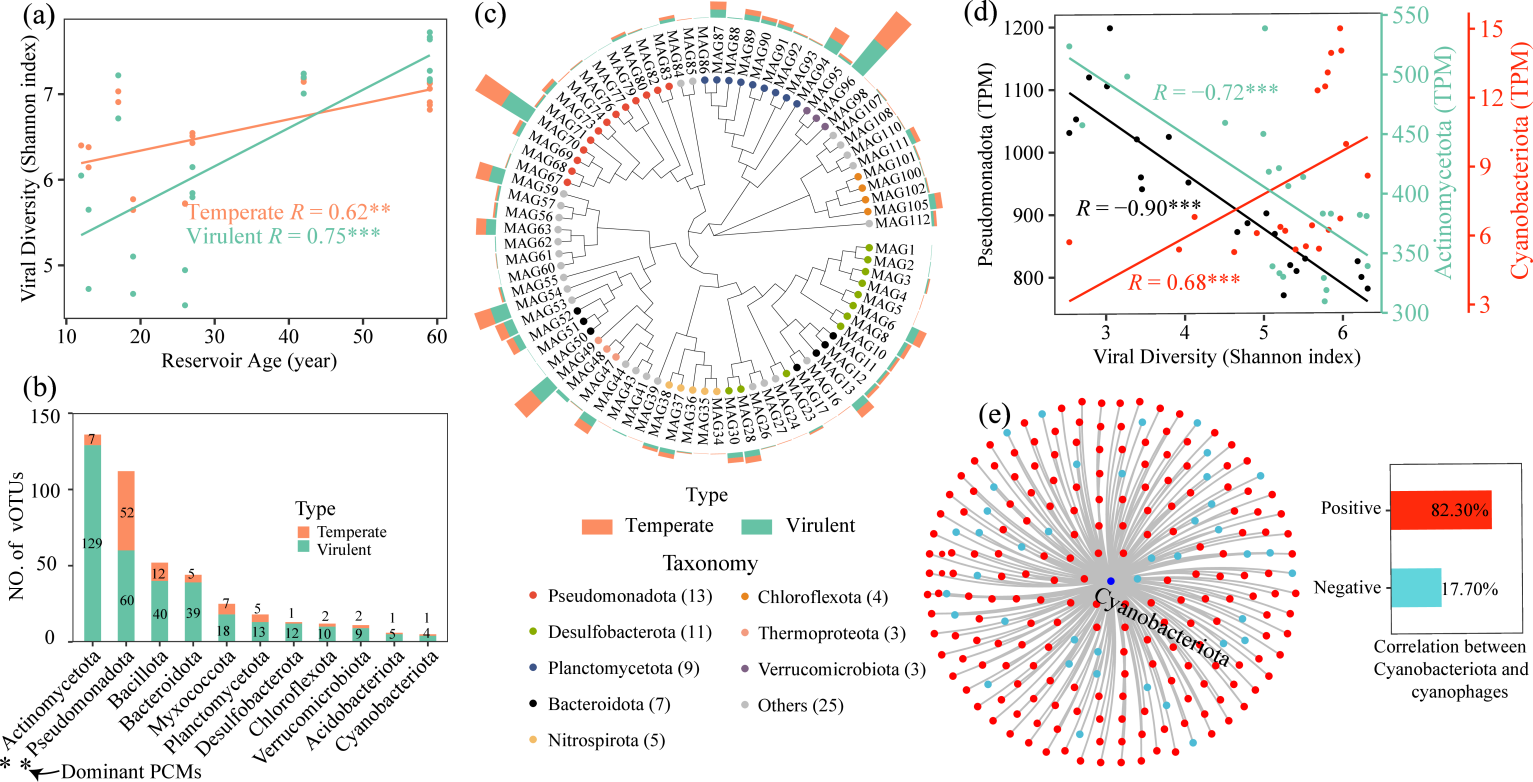
Viral community and host prediction. Correlation between viral α-diversity and reservoir age (**a**). Phylum-level hosts of viruses identified in the metatranscriptome (**b**). Eighty MAGs harboring P-cycling genes were predicted to serve as hosts for viruses, with bar height in the figure representing the number of vOTUs (**c**). Correlations between the abundances of Pseudomonadota, Actinomycetota, and Cyanobacteriota and the α-diversity of viruses for which these bacteria serve as hosts (**d**). The correlation between Cyanobacteriota abundance and that of cyanophages (**e**).

Both α- and β-diversity of temperate and lytic viruses were significantly positively correlated with increasing reservoir age (Fig. 4a ; Fig. S6c). Host prediction based on GTDB revealed that Pseudomonadota, Bacillota, Bacteroidota, Actinomycetota, Verrucomicrobiota, and Cyanobacteriota were the preferred hosts of diverse viruses (Fig. S7a). Among these phyla, Pseudomonadota and Actinomycetota served as hosts for the highest number of active viral taxa recovered from metatranscriptomes, whereas Cyanobacteriota ranked eleventh (Fig. 4b). At the genus level, *Streptomyces*, *Pseudomonas*, *Mycobacterium*, *Luteolibacter*, *Flavobacterium*, *Telluria*, *Collinsella*, *Pelagibacter*, *Sediminibacterium*, and *CAG-269* were the preferred hosts of viruses (Fig. S7b), with *Streptomyces*, *Nitrosarchaeum*, *Flavobacterium*, *Pseudomonas*, and *Pelagibacter* being the dominant infection targets of active viruses (Fig. S7c). MAG-based host prediction identified 80 MAGs that possessed P-cycling genes and served as viral hosts (Fig. 4c). These MAGs were distributed across 28 phyla, with Pseudomonadota being most abundant (13 MAGs), followed by Desulfobacterota and Planctomycetota. Specifically, 38 MAGs (47.50%) harbored at least *ppk1* or *ppk2* (Fig. 4c; Table S3), including *Methanoregula* (MAG44, MAG45), *Luteolibacter* (MAG95, MAG96), and *Cyanobium* (MAG110, MAG111). The abundance of *Luteolibacter* and *Cyanobium* was significantly positively correlated with reservoir age (Fig. S8a and c), as well as with the abundance of the viruses that infect them (Table S5; Fig. S8b and d).

The α-diversity of viruses infecting Pseudomonadota and Actinomycetota was significantly negatively correlated with the abundance of their respective hosts (Fig. 4d). Conversely, the α-diversity of viruses infecting Cyanobacteriota was significantly positively correlated with cyanobacterial abundance (Fig. 4d). Specifically, among the 226 vOTUs that targeted Cyanobacteriota, 186 (82.30 %) exhibited significant positive correlations between their abundances and the abundance of Cyanobacteriota (Fig. 4e). These cyanophages encoded genes associated with phosphorus-starvation responses, energy metabolism, and carbon fixation (Table S6).

**Fig 5:**
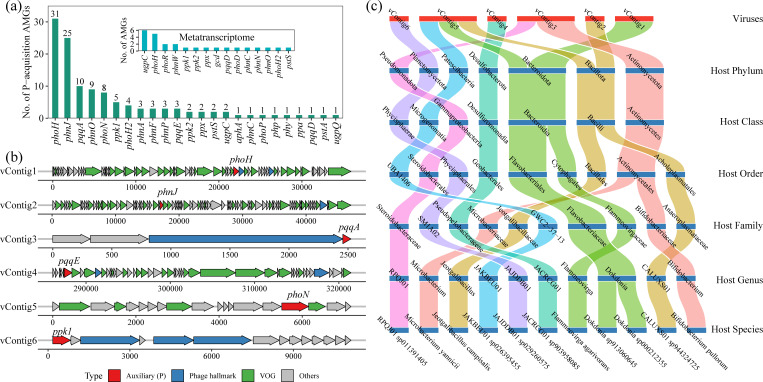
P-acquisition AMGs identified in sediments. Number of AMGs associated with P cycling detected in the metagenomic and metatranscriptomic data sets (**a**). Panel (**b**) depicts the genome organization of six representative vContigs, and panel (**c**) shows their predicted hosts.

We identified 29 types of virus-encoded P-cycling genes by integrating traditional sequence searches, advanced protein language models, and structural predictions (Fig. 5a). Specifically, *phoH* and *phnJ* were the most frequently detected, with a total of 31 and 25 gene sequences identified, respectivel*y*. The two viral contigs carrying these AMGs, vContig1 and vContig2, were confirmed to encode viral hallmark genes (Fig. 5b). Host prediction indicated that vContig1 and vContig2 likely infect hosts within Actinomycetota and Bacillota, respectively. Furthermore, we found that vContig5, which encodes *phoN*, may target a broad range of hosts spanning Patescibacteriota, Bacillota, and Bacteroidota. Virus-encoded P-cycling genes also included polyphosphate-accumulating genes (*ppk1*, *ppk2*), organic-phosphate-mineralizing genes (*phoN*, *phy*), inorganic-phosphate-solubilizing genes (*pqqA*, *pqqE*, *pqqD*), phosphonate-degradation genes (*phnA*, *phnC*, *phnF*, *phnP*), and the high-affinity inorganic phosphate transporter gene (*pstS*). Detailed information on the viruses encoding these genes, as well as their predicted hosts, is found in Table S4. Metatranscriptomic data further confirmed that active viruses encoded *ppk1*, *ppk2*, *pqqD*, *phoD*, *phnN*, *phoH*, and *pstS* (Fig. 5a). Furthermore, we found that the diversity of the AMGs mentioned above was significantly positively correlated with the diversity of PCMs (Fig. S9), although it was positively but not significantly correlated with reservoir age (Fig. S6d).

### Viral and physicochemical effects on P-cycling genes and microorganisms

**Fig 6:**
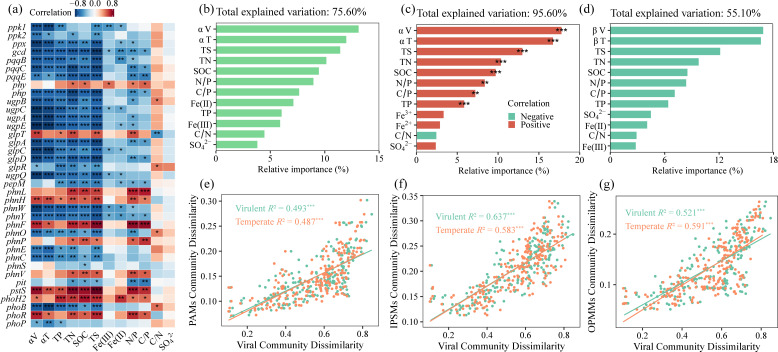
The relative importance of viral and physicochemical parameters to P-cycling genes and microorganisms. Correlations between P-cycling gene abundances and viral diversity or sediment physicochemical parameters. (**a**). Note: αV and αT represent the α-diversity of lytic and temperate viruses, respectively. This notation applies to all subsequent panels. Relative importance of viral α-diversity and physicochemical parameters in predicting P-cycling genes (**b**) and the α-diversity of PCMs (**c**). Relative importance of viral β-diversity and physicochemical parameters in predicting the beta diversity of PCMs (**d**). Note: βV and βT denote the β-diversity of lytic and temperate viruses, respectively. Correlations between β-diversity of PAMs, IPSMs, and OPMMs and viral β-diversity (**e–g**).

Correlation analyses revealed that the abundances of polyphosphate-accumulating genes (*ppk1*, *ppk2*), inorganic-phosphate-solubilizing genes (*gcd*, *pqqB*, *pqqC*, *pqqE*), and organic-phosphate-mineralizing genes (*php*, *ugpBCAE*, among others) were significantly negatively correlated with the α-diversity of lytic and temperate viruses, as well as with TP and TS contents (Fig. 6a). In contrast, the abundance of the *pstS* was significantly positively correlated with these variables. Hierarchical partitioning indicated that lytic viruses, temperate viruses, and TS were the primary drivers of changes in microbial P-cycling gene abundances (Fig. 6b). Hierarchical partitioning further showed that lytic viruses, temperate viruses, and TS were the dominant factors influencing both the α- and β-diversity of PCMs (Fig. 6c and d), all of which were significantly positively correlated with the α-diversity of PCMs (Fig. 6c). We separately compared the *R*² values from the regression analyses between the lytic viral community and each of the PAM, IPSM, and OPMM communities. The analysis revealed that the *R*² values for IPSMs and OPMMs with the lytic viral community were higher than those for PAMs (Fig. 6e through g).

The degree, negative cohesion, and stability of the co-occurrence networks were all significantly positively correlated with viral diversity (Fig. S11a, c and d). We calculated correlations between the abundances of PCMs and vOTUs and constructed co-occurrence networks. The number of PCMs-vOTU links was significantly positively correlated with the abundances of the former (Fig. S12a and c). The top 10 most highly connected taxa in the co-occurrence networks included *Streptomyces*, *Bradyrhizobium*, *Pseudomonas*, *Burkholderia*, *Aromatoleum*, and *Cupriavidus* (Fig. S12b through d).

## DISCUSSIONS

### Viral community enhances the diversity and community stability of PCMs

The α-diversity of PCMs increased with reservoir age (Fig. S3c), with viruses identified as a more important driver than any single chemical parameter (Fig. 6c). This result suggests that the viral community plays a key role in modulating PCMs in sediments. Host-prediction results indicated that dominant PCMs were preferentially infected by multiple viruses (Fig. 4b ; Fig. S7), which could reduce the abundances of these PCMs (Pseudomonadota, Actinomycetota; Fig. 4d). Several PCMs served as keystone taxa and exhibited high activity (Fig. 3a ; Fig. S4b), including *Streptomyces*, *Bradyrhizobium*, and *Burkholderia*, whose abundances declined with increasing reservoir age (Fig. 3b). However, the community diversity was increased along reservoir age gradients (Fig. S3c). This could be because viruses enhance the diversity of PCMs by lysing dominant (including keystone) taxa. Previous studies demonstrated that in soil pure-culture experiments, viral lysis of dominant microbial populations promoted the expansion of low-abundance taxa, thereby enhancing whole microbial diversity (26, 35).

Furthermore, similar to lytic viruses, temperate viruses exerted a positive influence on PCM diversity (Fig. 6c). This led us to hypothesize that this pattern might reflect a strategy where temperate viruses exert selective pressure on dominant PCM taxa. To test this, we analyzed host-encoded CRISPR spacers—the molecular memory of the prokaryotic adaptive immune system (CRISPR-Cas) that records past viral infections (36). The significant increase in CRISPR spacer density with reservoir age (Fig. S10c) provides independent support for the intensification of viral pressure (including from temperate phages) over time. Specifically, the rising viral pressure likely decreased the abundance of dominant PCM taxa, such as Pseudomonadota (Fig. 4b and d), thereby creating opportunities for the expansion of previously low-abundance species. Given that highly diverse microbial communities generally exhibit greater stability (37), our findings demonstrate significant positive correlations of PCM community stability with both viral diversity and reservoir age (Fig. S11d; Fig. 3d). This suggests that viruses play a key role in enhancing PCM stability with the increase in reservoir age.

### Preferential viral infection of PSMs over PAMs

With increasing reservoir age, the diversity of PAMs and OPMMs increased (Fig. 2c), whereas the diversity of IPSMs declined. Linear regression indicated that lytic viral communities explained the greatest proportion of variation in IPSMs, followed by OPMMs and then PAMs (Fig. 6e through g), suggesting that viral lytic pressure declined from IPSMs to OPMMs to PAMs. According to the “kill-the-winner” hypothesis, fast-growing microorganisms are more likely to be targeted by lytic viruses (38, 39). Previous studies have shown that microorganisms with smaller genomes tend to exhibit higher growth rates and possess fewer antiviral defense systems (40, 41). In this study, PAMs exhibited larger average genome sizes, more abundant antiviral defense systems, and longer doubling times than those of PSMs (Fig. S2c through e), implying that viruses preferentially infect PSMs. This is further supported by the narrower viral range in PAM genomes (Fig. S2f), enabling PAMs to maintain population growth despite viral pressure. Despite both being preferred viral hosts (Fig. S7), *Luteolibacter* (MAG96, Fig. 4c) increased in relative abundance with reservoir age (Fig. S8a), whereas *Streptomyces* exhibited a declining trend (Fig. 3b). *Luteolibacter* represents a potential novel polyphosphate-accumulating taxon, based on our functional annotation of key metabolic genes (*ppk1*, *ppx*, *pit*, *pstSABC*; Fig. 2d; Table S3) in its MAGs. This result could enhance the understanding of the functional diversity of *Luteolibacter*, although it has been widely detected in activated sludge, soil, marine, and freshwater systems (42–45). Collectively, viruses might preferentially infect PSMs, resulting in higher diversity of PAMs than that of PSMs (OPMMs and IPSMs; Fig. S2b) and leading to greater abundances of PA genes relative to IPS and OPM genes (Fig. S2a). Such host-selective differences provide a theoretical basis for directionally modulating microbial community functions via both “top-down” and “bottom-up” strategies.

### Viral lysis decreases abundances of polyphosphate-accumulating and phosphate-solubilizing genes

Although phosphorus content increased significantly with reservoir age (Fig. 1a), the abundances of microbial polyphosphate-accumulating, inorganic-phosphate-solubilizing, and most organic-phosphate-mineralizing genes declined markedly as phosphorus content rose (Fig. 6a). This pattern contrasted with previous reports that inorganic-phosphate-solubilizing and organic-phosphate-mineralizing genes were enriched in phosphorus-rich environments (46). Recent work has documented negative correlations between the abundances of *phnE* and *ppx* and phosphorus concentrations (47), partially aligning with our observations. Conversely, the abundance of *pstS* was significantly positively correlated with phosphorus content (Fig. 6a), in contrast to findings in the ocean, where *pstS* abundance decreases with increasing phosphorus concentrations (9). This discrepancy may imply that microbial phosphorus utilization becomes more efficient with the increase in reservoir age. Hierarchical partitioning revealed that lytic and temperate viruses, rather than sediment physicochemical variables, were the foremost factors driving changes in microbial P-cycling gene abundances (Fig. 6b; Fig. S10b). Correlation analyses further showed that the abundances of polyphosphate-accumulating, inorganic-phosphate-solubilizing, and most organic-phosphate-mineralizing genes declined as lytic viral diversity increased (Fig. 6a). Viral lysis pressure on dominant PCM taxa probably intensifies with the increase in reservoir age (Fig. 4b and d), likely accounting for the observed declines in the abundances of genes involved in PA, IPS, and OPM.

In this study, the influence of temperate viruses on the abundance of P-cycling genes is negative—paralleling the impact of lytic viruses (Fig. 6a). This negative influence may be linked to the overall increase in viral pressure on PCMs with reservoir age (Fig. S10c). For instance, the rising pressure could reduce the abundance of dominant PCM taxa (Pseudomonadota; Fig. 4b and d), thereby contributing to the observed decrease in P-cycling gene abundance. This pattern aligns with observations in other microbial systems, where increased investment in host defense systems is often associated with a reduction in the number of accessory functional genes, such as antibiotic resistance genes (41). Additionally, a previous study has reported that, to cope with viral pressure, microorganisms rapidly consume ATP to inhibit viral replication (48), which necessitates enhanced phosphorus acquisition to replenish the rapidly depleted ATP. This mechanism probably underpins the significant positive correlations observed between *pstS* abundance and both reservoir age and viral diversity (Fig. S1c; Fig. 6a). Collectively, our findings emphasize that escalating viral pressure with reservoir age could reshape microbial P-cycling processes.

### Viral enhancement of sediment P retention and release

In this study, we identified 29 types of AMGs responsible for P-cycling genes (Fig. 5a), exceeding the 17 types of AMGs recently reported from farmland, forest, Gobi desert, grassland, and mine-wasteland samples (49). Specifically, to the best of our knowledge, *phnJ* and *phy*-genes involved in OPM were discovered for the first time as virus-encoded AMGs. *phnJ* was detected 25 times and encodes the key enzyme for C-P bond cleavage in phosphonates, implying that viruses not only participate in phosphorus metabolism but also could be linked to methane production during this process (50). *ppk1* and *ppk2* are canonical markers of PAMs and have been reported as virus-encoded genes in phosphorus-rich environments (47, 51). Here, active viruses also carried *ppk1* and *ppk2* (Fig. 5a), suggesting that viral augmentation of microbial polyphosphate accumulation could enhance intracellular P sequestration and thereby help to mitigate eutrophication in reservoirs. *phoH* was detected 31 times in this study. As a phosphorus-starvation-response gene and one of the most widely distributed AMGs (32, 52), its prevalence underscores the pervasive role of viruses in modulating host phosphorus responses. Additionally, virus-encoded AMGs included genes for IPS (*pqqAED*), OPM (*phoN*), and high-affinity inorganic phosphate transport (*pstS*, *pstA*). Notably, the diversity of these AMGs was significantly positively correlated with the diversity of PCMs (Fig. S9), implying that virus-infected microorganisms carrying P-cycling genes could enhance the diversity of PCMs. Collectively, these results suggest that viruses serve as a crucial library of P-cycling genes in reservoir sediments and actively participate in microbial phosphorus retention and release. It is important to highlight that our findings on viral-encoded P-cycling genes are based on *in silico* analyses, and their functional roles await experimental confirmation.

### Potential virus-mediated cyanobacterial growth

Cyanobacterial blooms threaten water quality. Here, the relative abundance of cyanobacteria increased significantly with reservoir age (Fig. 2b). This rise might stem from viral lysis of dominant P-cycling taxa, which reduced their abundances and allowed the less abundant PCMs, such as Cyanobacteria, to proliferate (Fig. 4d). Moreover, cyanophages might promote the growth of Cyanobacteria by enhancing their ability to compete for phosphorus and carbon (Fig. 4d and e; Table S6), since these viruses encode AMGs that participate in phosphorus-starvation responses (*phoH*), energy metabolism, and carbon-fixation modules within KEGG (Table S6), including genes of the pentose phosphate pathway, heme biosynthesis, photosystem II, and the dicarboxylate-hydroxybutyrate cycle. This was consistent with the report that cyanobacteria could maintain a competitive advantage even under CO₂(aq)-limited conditions (53).

Recent studies using short-term viral addition experiments have confirmed that viral lysis of prokaryotic cells releases labile dissolved organic matter (DOM) (30), which indirectly indicates the concomitant release of bioavailable phosphorus since it is also an essential component of microbial cells. However, directly quantifying viral impacts on phosphorus cycling by viral addition could be a significant challenge because labile phosphate is usually rapidly reassimilated by the microbial community, as evidenced by cyanobacterial blooms under low dissolved phosphorus conditions (54, 55). Given the frequent material exchange between surface sediments and the overlying water column (5, 6), the reservoir age-dependent viral lysis of dominant PCMs (Fig. 2b ,4b and d) may supply labile P to the water. This could promote cyanobacterial growth and potentially contribute to algal bloom outbreaks.

### Conclusion

We showed that both the community structure of PCMs and the abundances of P-cycling genes in reservoir sediments changed systematically along the reservoir age. Viruses emerged as the principal drivers of changes in the diversity of PCMs and P-cycling gene abundances. Viruses that preferentially lysed dominant PCMs may reduce the phosphorus-solubilizing and polyphosphate-accumulating potential of the microbial community, while stimulating high-affinity inorganic phosphate transport. Additionally, viruses appeared to directly participate in phosphorus cycling via harboring and actively expressing numerous P-cycling genes. Finally, the findings revealed that viral activity promoted an increase in cyanobacterial abundance along reservoir age. Collectively, our results highlight how sediment-associated viruses shape P-cycling microbial communities and gene abundances, deepening our insight into viral control of biogeochemical cycling and emphasizing that viruses should be incorporated in future reservoir eutrophication management.

## MATERIALS AND METHODS

### Site description and sediment collection

The Wujiang River is the largest southern tributary of the upper reaches of the Yangtze River. Its geographical coordinates range from 25°56′ to 30°22′ N and 104°10′ to 109°12′ E. The total length is 1,037 km, its drainage area is 88,267 km², of which 67,500 km² is located within Guizhou Province. Approximately 87% of the Wujiang basin consists of plateaus and mountains, forming a typical canyon-type river. The annual mean air temperature is 14°C, and the mean annual precipitation is 1,195 mm. Reservoirs in the Wujiang basin were constructed gradually since the 1960s (Table S1).

We collected surface sediments (0–5 cm in depth) from nine reservoirs using transparent polycarbonate tubes 60–120 cm in length and 63 mm in diameter; three to six sediment cores were designed to be taken per reservoir. Immediately after collection, samples were transported vertically to the laboratory. Subsequently, the sediment samples were stratified and homogenized, then divided into two portions: one for physicochemical analyses, and the other stored at −80°C for future DNA sequencing. The sediment samples used for metatranscriptomic sequencing were flash-frozen in liquid nitrogen immediately after collection.

### Determination of sediment physicochemical properties

After thorough homogenization, surface sediment samples (0–5 cm in depth) were acidified, rinsed with deionized water, freeze-dried, and analyzed with an elemental analyzer for SOC, TN, and TS. Pore-water aliquots were filtered through 0.22 µm membranes and analyzed for sulfate (SO_4_^2−^) by anion chromatography. For Fe(III) and Fe(II), 0.5 mL of homogenized sediment (exact mass recorded) was extracted with 4.5 mL of 0.5 mol L^−1^ HCl and quantified spectrophotometrically (56). TP in sediments was determined using the SMT protocol (57).

### Metagenomic and metatranscriptomic sequencing and sequence processing

Omics sequencing was prepared for surface sediments. Metagenomic sequencing of extracted sediment DNA was conducted on the Illumina NovaSeq platform by Shanghai Majorbio Bio-pharm Technology Co., Ltd. For metatranscriptomic data, RNA integrity and purity were verified by agarose gel electrophoresis and the Agilent 5400 Fragment Analyzer (Agilent, Santa Clara, CA, USA). Ribosomal RNA was depleted from total RNA, and the remaining mRNA was fragmented and reverse-transcribed into cDNA. Libraries were sequenced on the Illumina PE150 platform by Novogene Co., Ltd. (Tianjin, China). Detailed methodologies for the metagenomic and metatranscriptomic analyses are provided in the Supplementary Material. Based on the taxonomic classifications, we parsed the lineage information for all sequences harboring P-cycling genes and subsequently categorized the corresponding microbial taxa into three functional guilds: PAMs, IPSMs, and OPMMs.

### Viral contig identification and calculation of abundances

Contigs shorter than 2,000 bp were removed with SeqKit v2.6.1 (58). The remaining sequences were processed to identify viral information as follows: potential viral sequences were initially identified using geNomad v1.8.1 (score > 0.95), VirSorter2 v2.2.4 (score > 0.95), VirFinder v1.1 (*P* < 0.05), and CheckV v1.0.3 (virus gene num ≥1) (59–61). Viral proteins were predicted with prodigal-gv v2.11.0 (https://github.com/apcamargo/prodigal-gv), and viral-like genes were identified with HMMER (http://hmmer.org/) against the Virus Orthologous Groups Database (https://vogdb.org/), retaining only hits with an E-value ≤ 0.00001. Integrating these outputs yielded vOTUs for each sample. Non-redundant vOTUs were then obtained by dereplication at the species level using the ANI-based clustering pipeline provided in MGV (https://github.com/snayfach/MGV/tree/master/ani_cluster; 95% ANI-average nucleotide identity, 85% AF-aligned fraction). Taxonomic assignments of vOTUs were performed with geNomad. Abundances of vOTUs were calculated using the contig module of CoverM.

### Identification of virus-encoded P-cycling genes

To maximize the likelihood of identifying virus-encoded AMGs, we integrated three complementary approaches: (i) Viral protein sequences were structurally predicted with ESMFold (GPU-accelerated) (62), followed by functional annotation via Foldseek against the Swiss-Prot database (63). (ii) The protein-language-model-based tool PLMsearch was employed for direct functional annotation of viral proteins (64); results with similarity thresholds < 0.8 were removed. (iii) Viral proteins were functionally annotated against the KEGG database via KofamKOALA; hits with E-values > 0.0001 were excluded. Finally, the outputs from all three methods were consolidated and cross-referenced with the databases of VIBRANT and DRAM-v (65, 66).

### Viral protein-sharing network, host prediction, and life-cycle inference

vConTACT2 v0.11.3 (default parameters) was employed to construct a protein-sharing network between the viruses obtained in this study and the NCBI Prokaryotic Viral RefSeq v201 database (67), and the network was visualized by Cytoscape v3.10.3. Subsequently, GTDB and the MAGs recovered in this study were merged, and the hosts of the vOTUs were predicted using PHIST v1.2.1, which achieves a favorable balance between performance and accuracy for environmental metagenomic data sets (68, 69). The life cycle of each vOTU, including lytic and lysogenic styles, was predicted with PhaTYP (70).

### Statistical analysis

Both α-diversity (Shannon index) and β-diversity for viruses and PCMs were calculated using the vegan package in R. Co-occurrence networks of PCMs were constructed based on significant correlations (|*r*| > 0.7, *P* < 0.05) calculated using the Hmisc package. Subgraphs were then extracted from these networks using the *induced_subgraph* function from the igraph package, and node degrees within the subgraphs were calculated with the *degree* function. To clarify, the sub-networks were derived through a two-step process: First, a global co-occurrence network was inferred based on genus-level correlations across all samples. Then, for each individual sample, a sub-network was extracted from this global network using the *subgraph* function in the igraph package, retaining only the nodes (genera) present in that sample and all edges connecting them. For each sample-specific sub-network, we calculated its network cohesion, a metric comprising separate positive and negative cohesion components that quantify the overall strength of positive and negative co-occurrence associations within the local community, respectively (71). The ratio of negative to positive cohesion was used as an indicator of community stability (72). The phylogenetic tree of prokaryotic MAGs was constructed using the infer module of GTDB-Tk, with a set of 53 archaeal and 120 bacterial marker genes for multiple sequence alignment. Phylogenetic trees of MAGs were visualized with ggtree (73). The relative contributions of viruses and sediment physicochemical variables to P-cycling pathways and microbial diversity were assessed using the *rdacca.hp* function in R. This method allows for the quantification of independent versus shared variation, enabling the disentanglement of their individual impacts (74). Unless otherwise specified, all correlations reported herein were Spearman rank correlations. All figures were generated with the ggplot2 package in R unless otherwise stated.

## Data Availability

The omics data have been deposited in the NCBI under BioProject accession number PRJNA761326.

## SUPPLEMENTAL MATERIAL

Supplemental material aem.02481-25-s0001.docx Supplemental methods; Fig. S1 to S12.

Supplemental tables aem.02481-25-s0002.xlsx Tables S1 to S7.

ASM does not own the copyrights to Supplemental Material that may be linked to, or accessed through, an article. The authors have granted ASM a non-exclusive, world-wide license to publish the Supplemental Material files. Please contact the corresponding author directly for reuse.
