# Erratum for Wu et al., “Lytic viruses drive the decrease in polyphosphate-accumulating and phosphate-solubilizing potential of microbial communities with increasing reservoir age”

**DOI:** 10.1128/aem.01122-26

**Published:** 2026-06-18

**Authors:** Qiusheng Wu, Debin Wu, Jiayi Wang, Heng Wang, Jingjing Peng, Yuan Zhao, Jingan Chen, Quan Yuan

**Affiliations:** 1 State Key Laboratory of Environmental Geochemistry, Institute of Geochemistry, Chinese Academy of Sciences 85408, Guiyang, China; 2 University of Chinese Academy of Sciences 74519, Beijing, China; 3 State Key Laboratory of Nutrient Use and Management, College of Resources and Environmental Sciences, China Agricultural University 587661, Beijing, China; 4 Guizhou Province Field Scientific Observation and Research Station of Hongfeng Lake Reservoir Ecosystem, Guiyang, China

## ERRATUM

Volume 92, no. 5, e02481-25, 2026, https://doi.org/10.1128/aem.02481-25. The affiliations and the affiliation numbers for each author should appear as shown in this erratum.

