# Supplementary material for: Lytic viruses drive the decrease in polyphosphate-accumulating and phosphate-solubilizing potential of microbial communities with increasing reservoir age
Source: Appl Environ Microbiol. 2026 Apr 27;92(5):e02481-25. doi: 10.1128/aem.02481-25 (PMC13188868; doi:10.1128/aem.02481-25)
Supplement: Supplemental material — Supplemental methods; Fig. S1 to S12. [file aem.02481-25-s0001.docx]

**Supplemental materials**

**Lytic viruses drive the decrease of polyphosphate-accumulating and phosphate-solubilizing potential of microbial communities with increasing reservoir age**

Qiusheng Wu^1,2^, Debin Wu^1^, Jiayi Wang^1^, Heng Wang^1,2^, Jingjing Peng^3^, Yuan Zhao^1^, Jingan Chen^1,4,^*, Quan Yuan^1,4,^*

^1^State Key Laboratory of Environment Geochemistry, Institute of Geochemistry, Chinese Academy of Science, Guiyang 550081, China

^2^University of Chinese Academy of Sciences, Beijing 100049, China

^3^State Key Laboratory of Nutrient Use and Management, College of Resources and Environmental Sciences, China Agricultural University, Beijing 100193, China

^4^Guizhou Province Field Scientific Observation and Research Station of Hongfeng Lake Res-ervoir Ecosystem, Guiyang, 551499, PR China

**Supplementary Methods:** **Metagenomic and Metatranscriptomic sequencing and sequence processing**

For metagenomic data, raw reads were quality-controlled with fastp v0.23.4 to generate clean data(1), and the clean data were assembled into contigs using MEGAHT v1.2.9 (2). Prodigal (-meta) v2.6.3 was employed to predict open reading frames (ORFs) (3), after which MMseqs2 v15.6f452 (--min-seq-id 0.95 -c 0.9 --cov-mode 1 --cluster-mode 2) was used to dereplicate genes across all samples to construct a non-redundant gene catalog (4). Clean reads were mapped to the non-redundant gene catalog with BWA v0.7.18 (5), and the resulting alignments were processed with SAMtools v1.20 to obtain ORF abundances (6). To account for gene length and sequencing depth biases, ORF abundance was quantified as Transcripts Per Kilobase of exon model per Million mapped reads (TPM). Functional annotation was performed against KEGG via KofamKOALA (7), to identify and quantify P-cycling genes (Table S2). Taxonomic assignment of the non-redundant gene catalog was carried out with Kraken2 (8) against the RefSeq database.

For metatranscriptomic data, raw reads were quality-filtered with fastp, and mRNA reads were isolated with SortMeRNA (9). Subsequent assembly, ORF prediction, functional annotation, and taxonomic assignments followed the same pipeline as the metagenomic data.

To recover metagenome assembled genomes (MAGs), contigs were binned within MetaWRAP v1.3.2 using MetaBAT 2, MaxBin 2, and CONCOCT (10). The bin_refinement module was then used to integrate the three binning results, and CheckM2 v1.0.2 was applied to assess completeness and contamination (11). Those MAGs with < 50 % completeness or > 10 % contamination were discarded. Dereplication of metagenome-assembled genomes (MAGs) was performed using skDER v1.2.8 (https://github.com/raufs/skDER). Given the potential impact of completeness and contamination estimates on genome size, we calibrated the genome sizes following a previously established method (12). Taxonomic classification of MAGs was carried out with GTDB-Tk v2.4.0 and the GTDB database release R260 (13). MAG abundance and growth rate index were calculated using CoverM v0.7.0 (https://github.com/wwood/CoverM) and gRodon2 (https://github.com/jlwecoevo/gRodon2), respectively. Functional annotation of MAGs against KEGG via KofamKOALA identified 112 MAGs containing phosphorus-cycling genes (Table S3). DefensePredictor (https://github.com/PeterDeWeirdt/defense_predictor) was applied to screen for anti-phage defense systems in the MAGs.


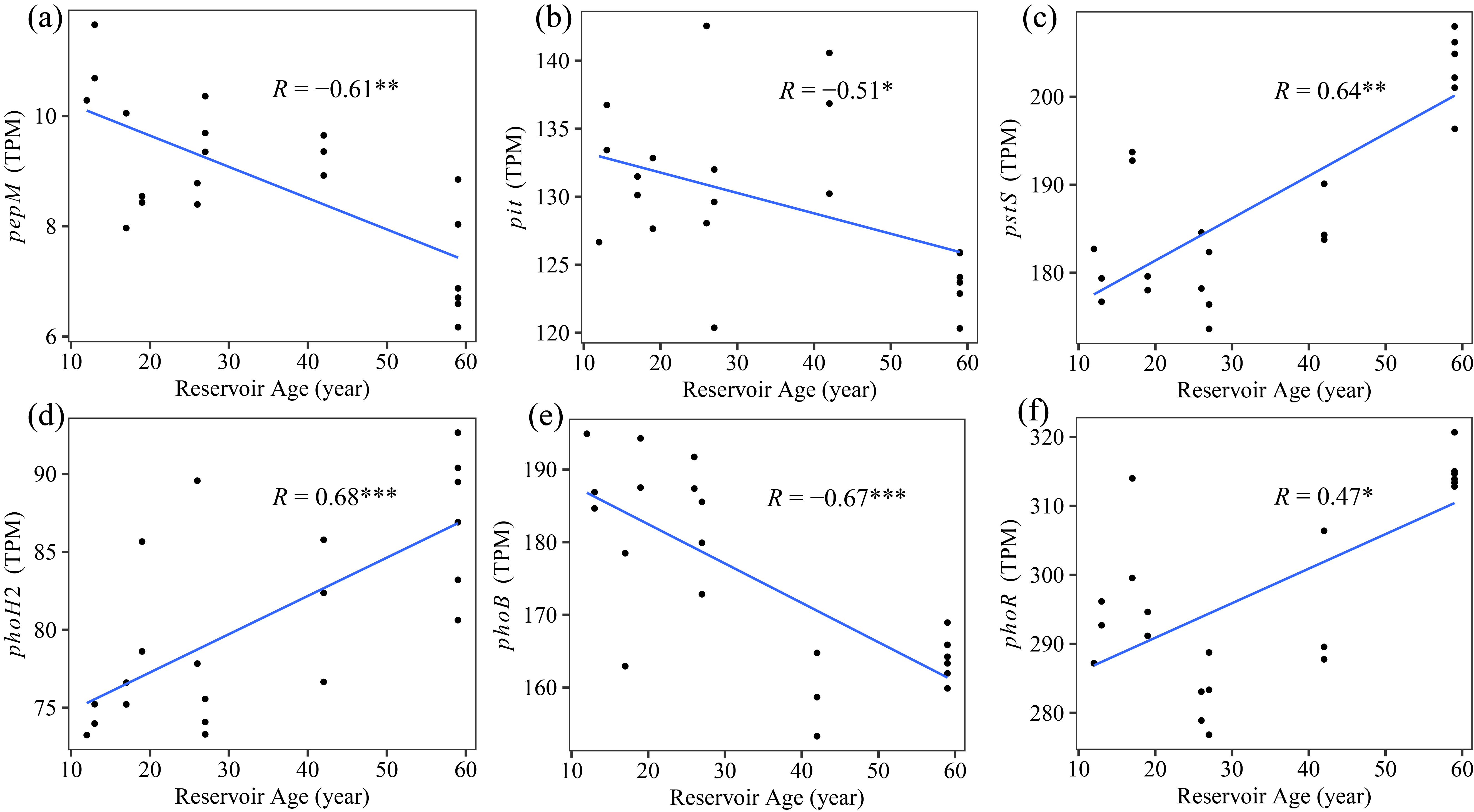


**FIG. S1** Relationship between P-cycling gene profiles and reservoir age


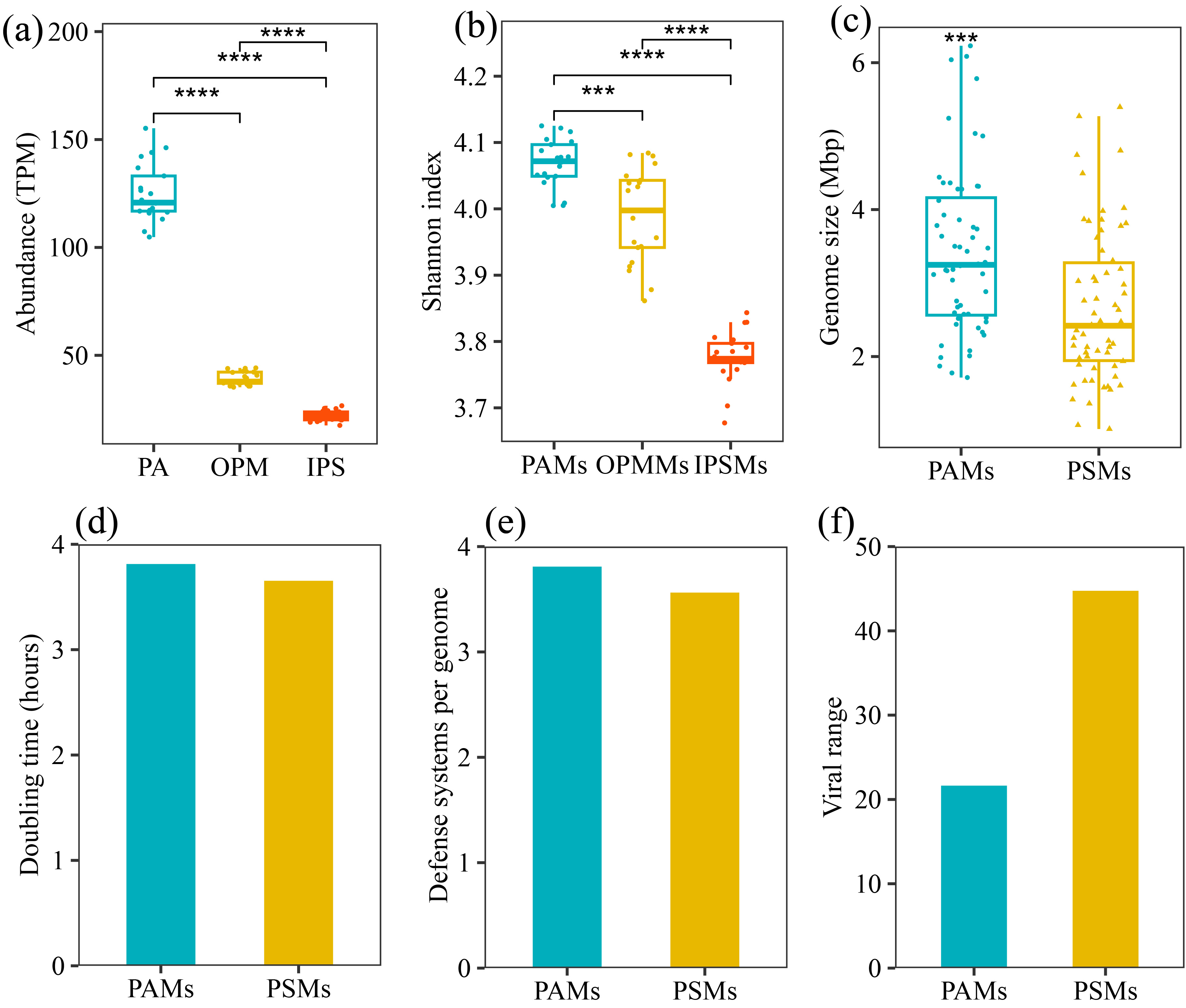


**FIG. S2** Comparative functional and genomic features of polyphosphate-accumulating microorganisms (PAMs) and phosphate-solubilizing microorganisms (PSMs). Differences in the abundance of phosphorus-cycling pathways among polyphosphate accumulation (PA), organic phosphate mineralization (OPM), and inorganic phosphate solubilization (IPS; a). Differences in the α-diversity of PAMs, inorganic-phosphate-solubilizing microorganisms (IPSMs), and organic phosphate-mineralizing microorganisms (OPMMs; b). Differences in genome size, doubling time, antiviral defense systems and viral range between PAMs and PSMs(c-f).


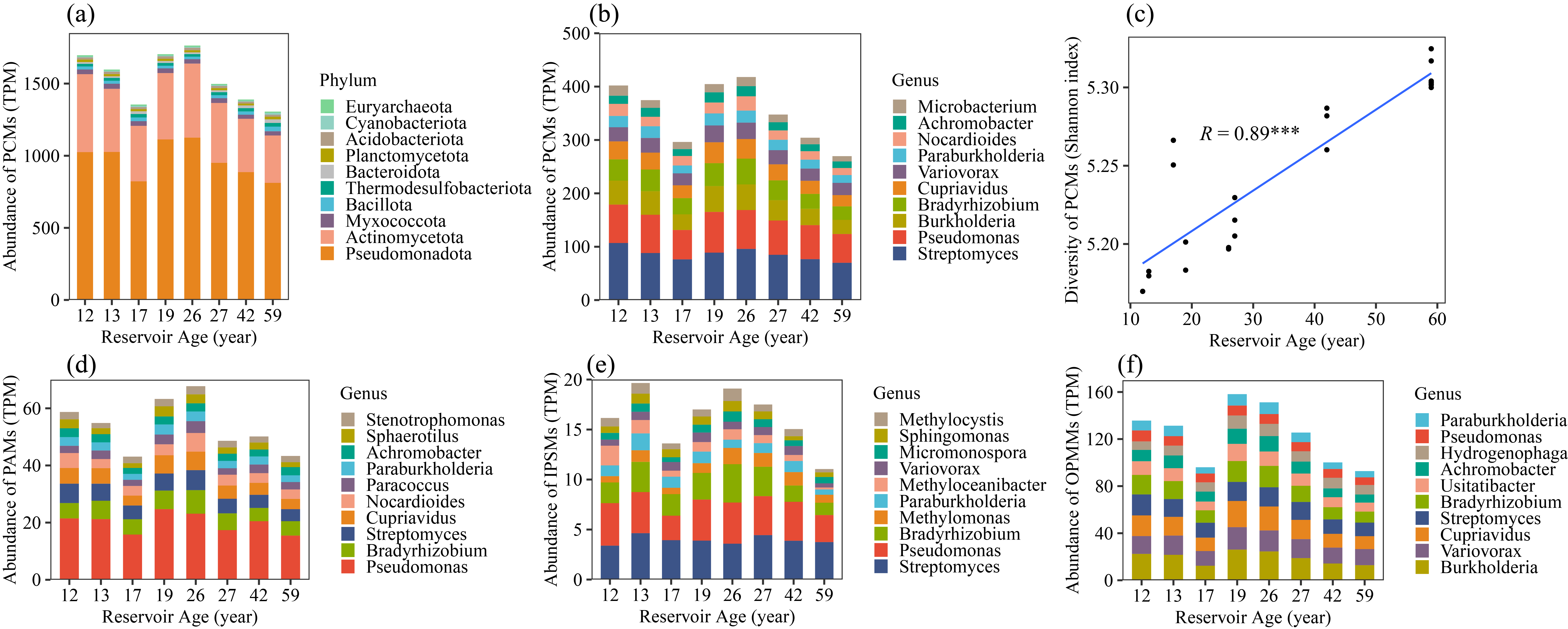


**FIG. S3** P-cycling microbial composition and diversity in sediments. Top 10 most abundant phosphorus-cycling microorganisms (PCMs) at the phylum and genus levels (a-b). Correlation between the α diversity of PCMs and reservoir age (c). d-e shows top 10 most abundant polyphosphate-accumulating microorganisms (PAMs), inorganic-phosphate-solubilizing microorganisms (IPSMs), and organic-phosphate-mineralizing microorganisms (OPMMs) .


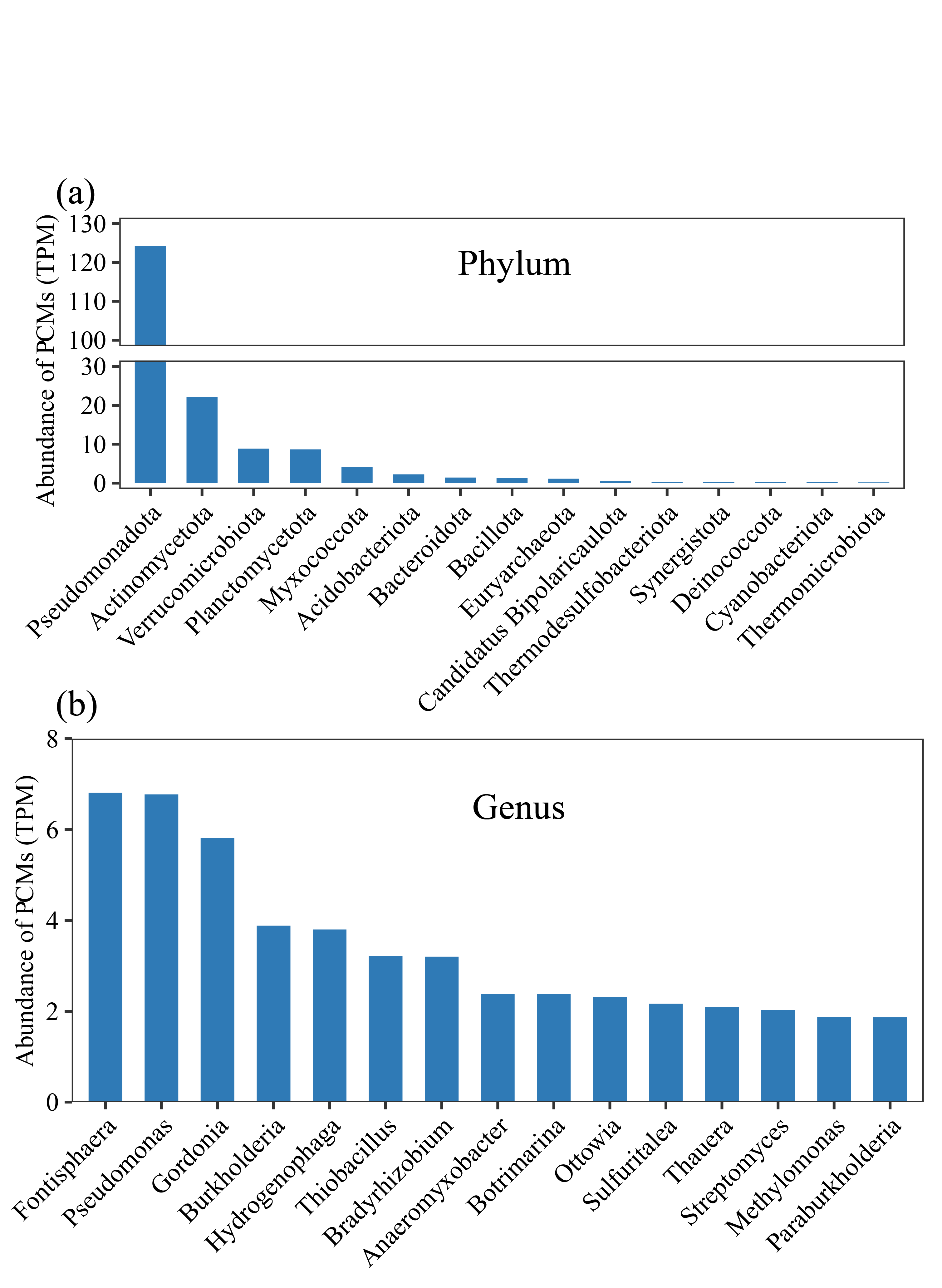


**FIG. S4** Top 15 most abundant phosphorus-cycling microorganisms (PCMs) at the phylum and genus levels in the metatranscriptome.


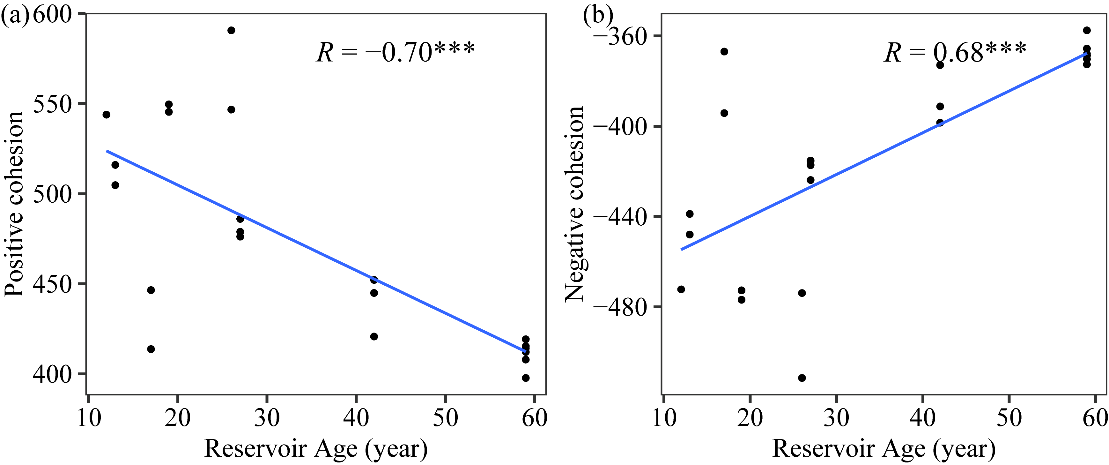


**FIG.** S5 The topological features of phosphorus-cycling microorganisms (PCMs).


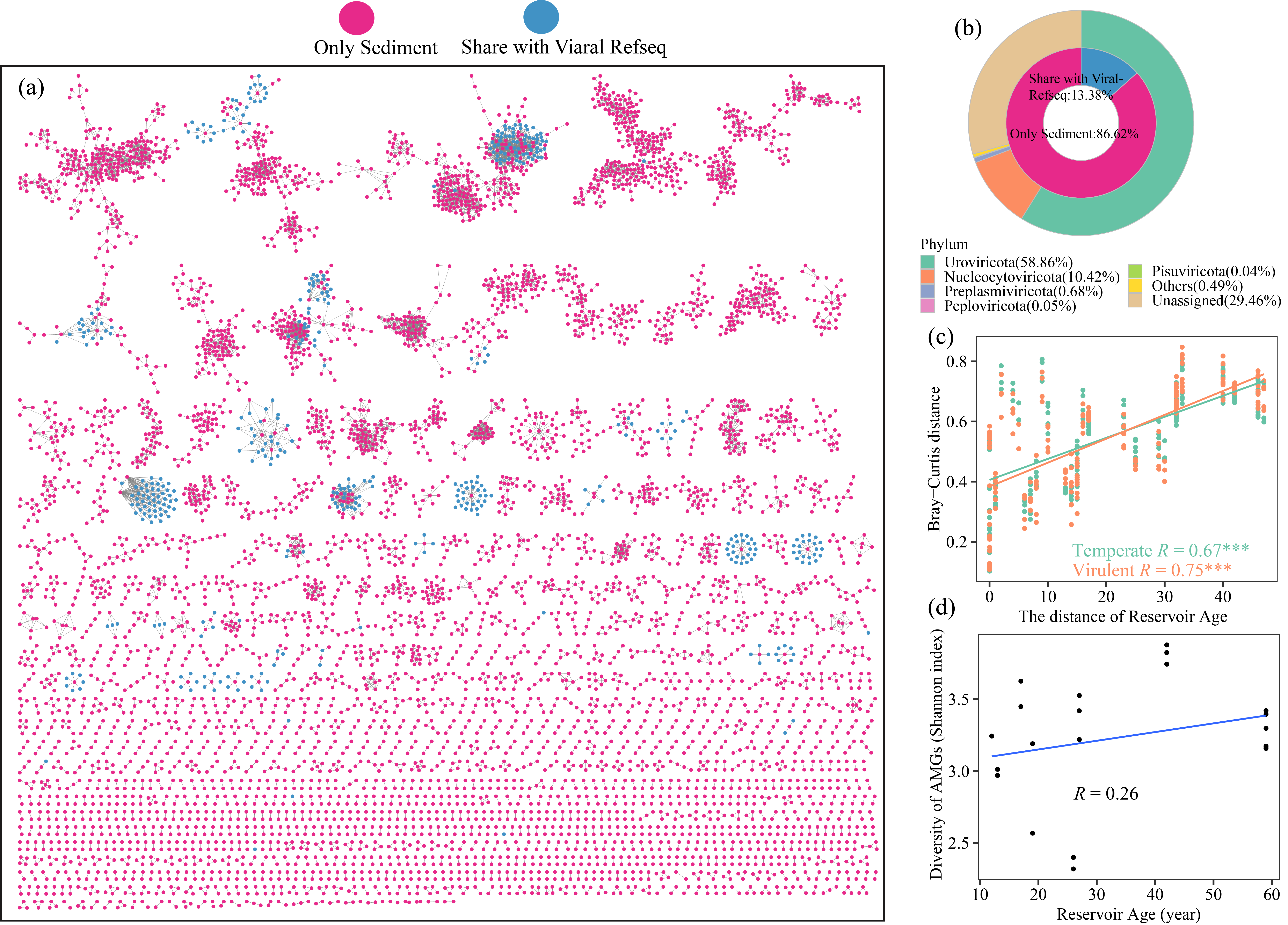


**FIG. S6.** Taxonomic diversity of viruses in sediments. Gene-sharing network of viruses from sediments and RefSeq prokaryotic viruses (a). Pie graph showing the relative proportion of shared viral clusters between sediments and RefSeq prokaryotic viruses, and relative proportion and taxonomic classification of vOTUs based on the latest ICTV classification using geNomad v1.8.1 (b). Correlations among viral communities, the α-diversity of P-cycling AMGs, and the reservoir age(c-d).


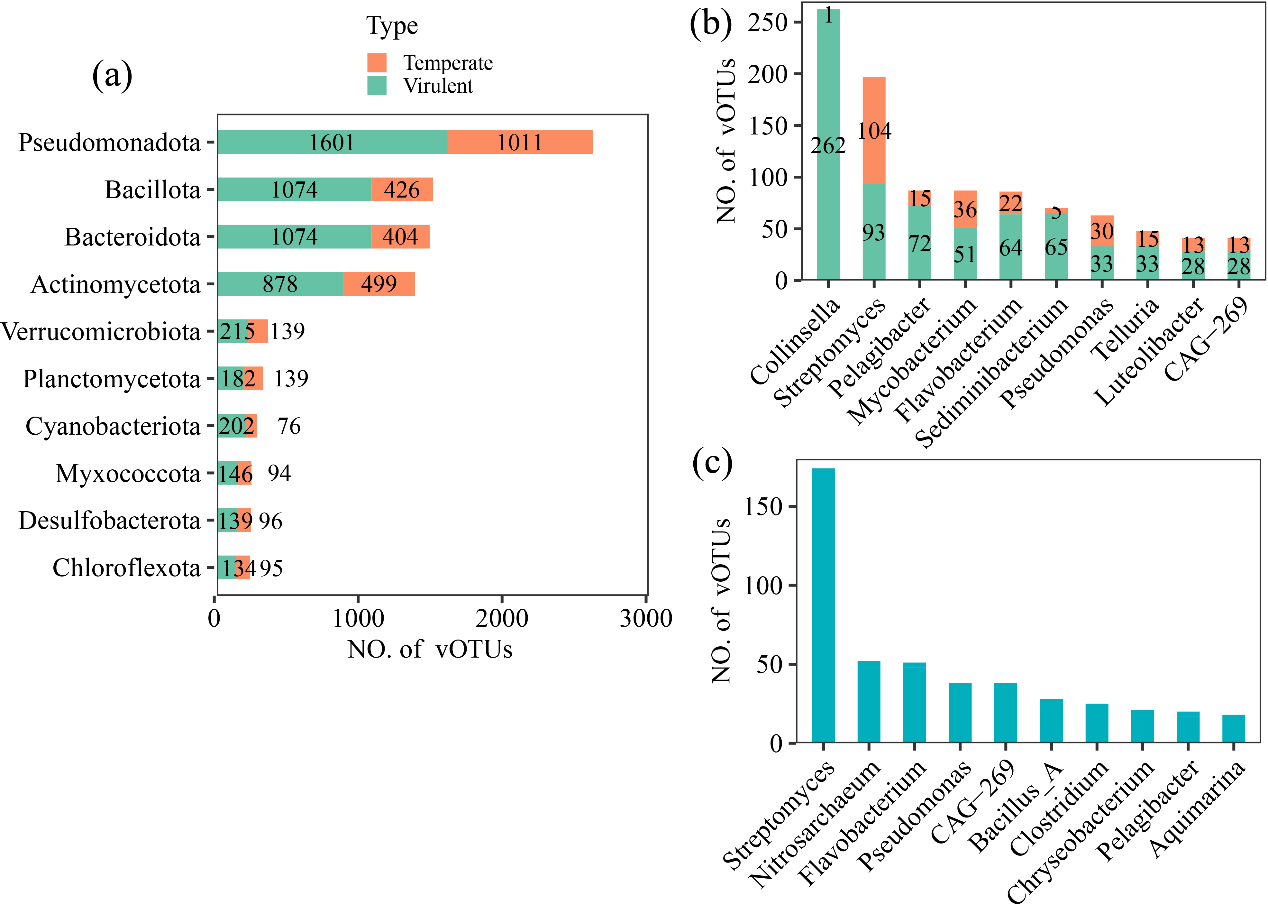


**FIG. S7** Phylum-level hosts of viruses identified in the metagenome (a). Genus-level hosts of viruses identified in the metagenome (b) and metatranscriptome (c).


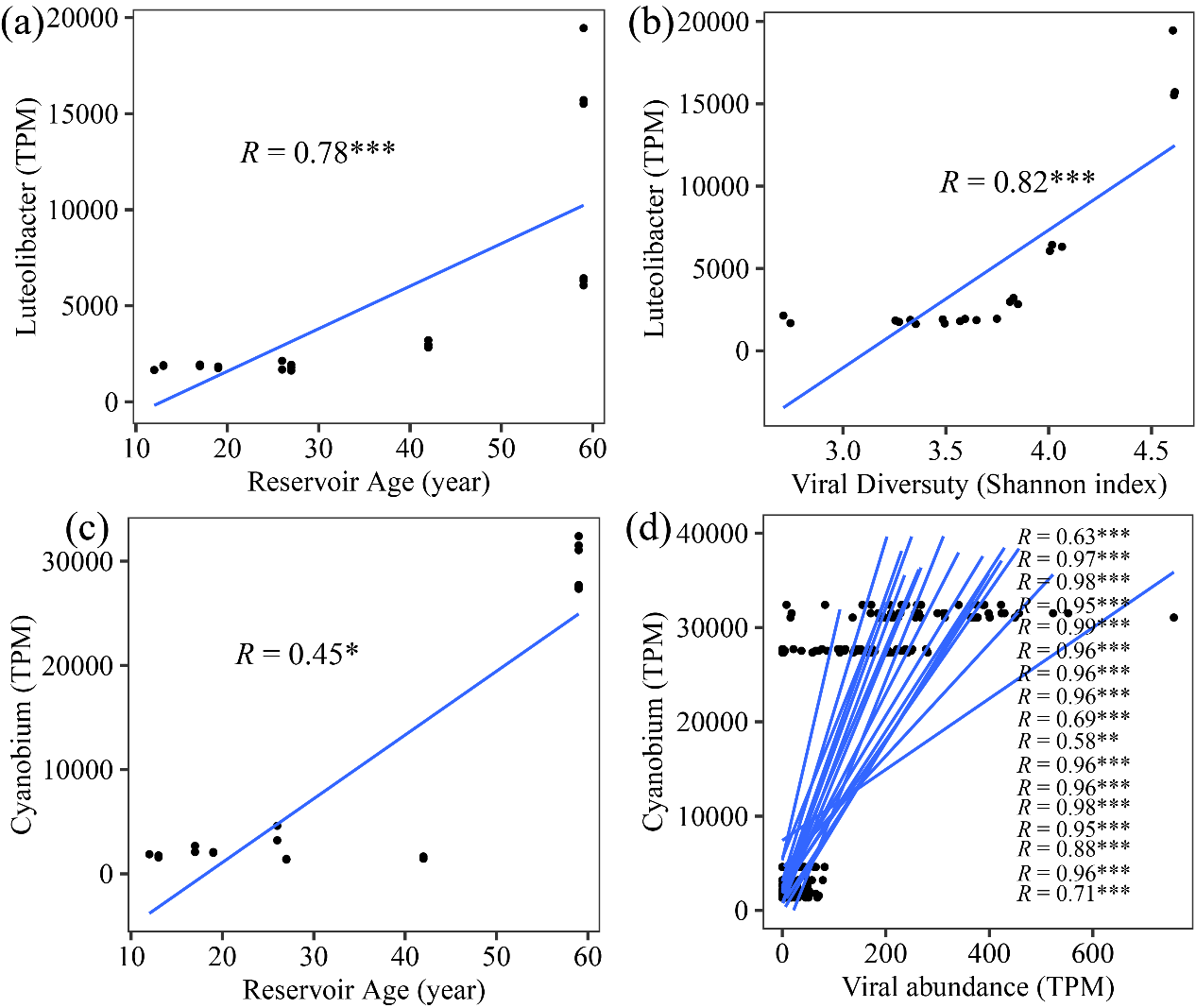


**FIG. S8.** Relationships between the abundance of key MAGs and reservoir age, and the abundance or diversity of their associated viruses.


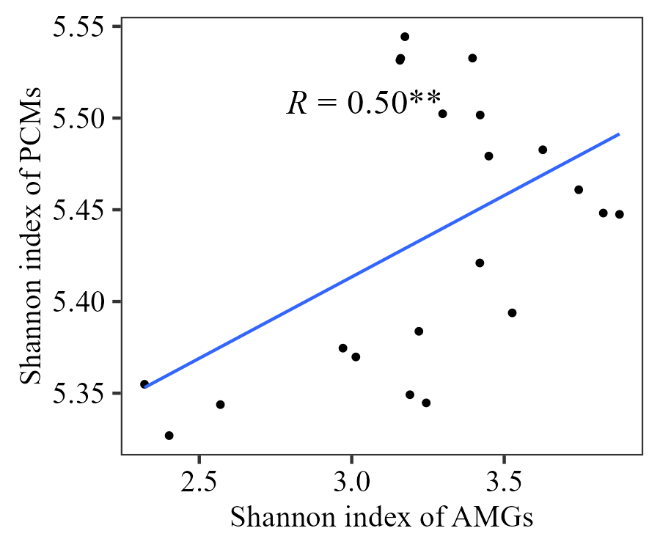


**FIG. S9.** The relationships between phosphorus-cycling microorganisms (PCMs) and auxiliary metabolic genes (AMGs) associated with phosphorus cycling.


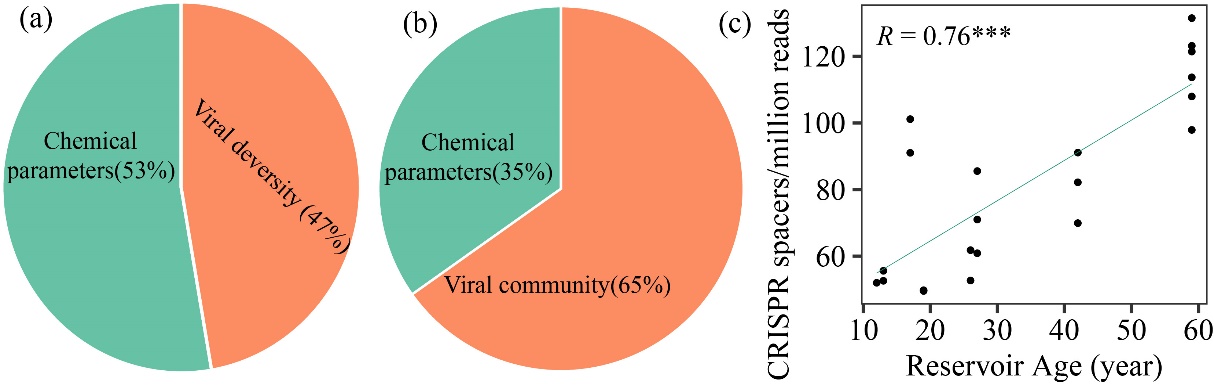


**FIG. S10** The relative importance of viral and physicochemical parameters to the diversity of PCMs (a) and the abundance of P-cycling genes (b). The correlation between CRISPR spacer density and reservoir age (c).


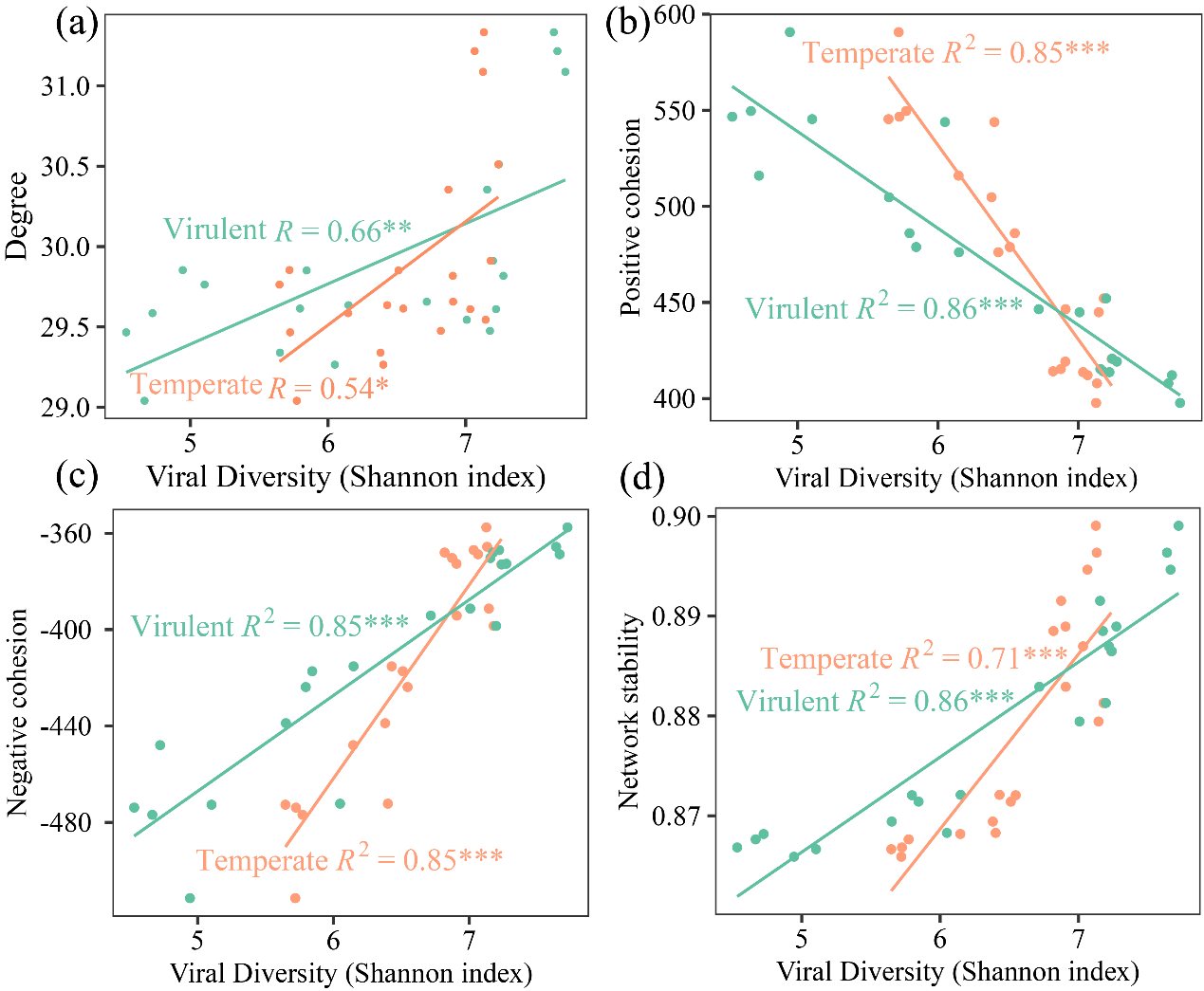


**FIG. S11.** The relationships between key topological parameters of the network and viral diversity.


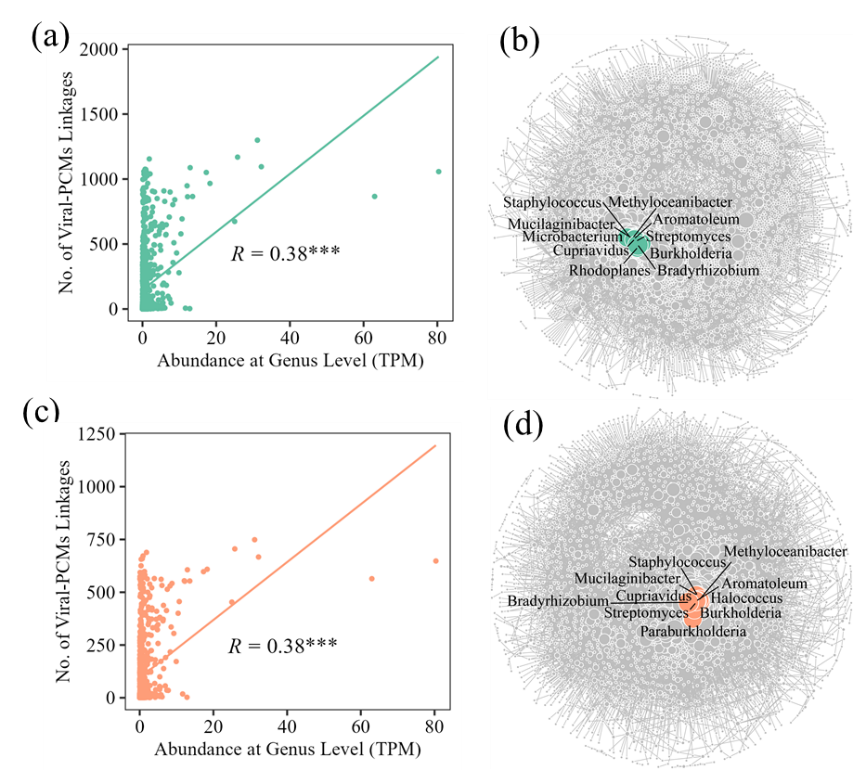


**FIG. S12.** **The relationships between phosphorus-cycling microorganisms (PCMs) and viruses**. Number of linkages between lytic viruses and PCMs (a). Top 10 PCMs with the highest degree of lytic virus linkages (b). Number of linkages between temperate viruses and PCMs (c). Top 10 PCMs with the highest degree of temperate virus linkages (d).

**References**

1. Chen S, Zhou Y, Chen Y, Gu J. 2018. fastp: an ultra-fast all-in-one FASTQ preprocessor. Bioinformatics 34:i884-i890.

2. Li D, Liu C-M, Luo R, Sadakane K, Lam T-W. 2015. MEGAHIT: an ultra-fast single-node solution for large and complex metagenomics assembly via succinct de Bruijn graph. Bioinformatics 31:1674-1676.

3. Hyatt D, Chen G-L, LoCascio PF, Land ML, Larimer FW, Hauser LJ. 2010. Prodigal: prokaryotic gene recognition and translation initiation site identification. BMC Bioinformatics 11:119.

4. Steinegger M, Söding J. 2017. MMseqs2 enables sensitive protein sequence searching for the analysis of massive data sets. Nat Biotechnol 35:1026-1028.

5. Li H, Durbin R. 2009. Fast and accurate short read alignment with Burrows–Wheeler transform. Bioinformatics 25:1754-1760.

6. Danecek P, Bonfield JK, Liddle J, Marshall J, Ohan V, Pollard MO, Whitwham A, Keane T, McCarthy SA, Davies RM, Li H. 2021. Twelve years of SAMtools and BCFtools. GigaScience 10:giab008.

7. Aramaki T, Blanc-Mathieu R, Endo H, Ohkubo K, Kanehisa M, Goto S, Ogata H. 2020. KofamKOALA: KEGG Ortholog assignment based on profile HMM and adaptive score threshold. Bioinformatics 36:2251-2252.

8. Wood DE, Lu J, Langmead B. 2019. Improved metagenomic analysis with Kraken 2. Genome Biology 20:257.

9. Kopylova E, Noé L, Touzet H. 2012. SortMeRNA: fast and accurate filtering of ribosomal RNAs in metatranscriptomic data. Bioinformatics 28:3211-3217.

10. Uritskiy GV, DiRuggiero J, Taylor J. 2018. MetaWRAP—a flexible pipeline for genome-resolved metagenomic data analysis. Microbiome 6:158.

11. Chklovski A, Parks DH, Woodcroft BJ, Tyson GW. 2023. CheckM2: a rapid, scalable and accurate tool for assessing microbial genome quality using machine learning. Nat Methods 20:1203-1212.

12. von Meijenfeldt FAB, Hogeweg P, Dutilh BE. 2023. A social niche breadth score reveals niche range strategies of generalists and specialists. Nature Ecology & Evolution 7:768-781.

13. Chaumeil P-A, Mussig AJ, Hugenholtz P, Parks DH. 2020. GTDB-Tk: a toolkit to classify genomes with the Genome Taxonomy Database. Bioinformatics 36:1925-1927.
